# Working with Mike

**DOI:** 10.1098/rspa.2022.0168

**Published:** 2022-03

**Authors:** Steven M. Christensen

**Affiliations:** Department of Physics and Astronomy, University of North Carolina at Chapel Hill, Chapel Hill, NC, USA

During the late 1970s and early 1980s, I worked with Mike Duff on seven papers [1–7]. In the process, I came to admire and learn from him in ways I have always valued. In other papers [8,9] he and I have already told various stories about working together. Sharing my memories of what he was like as a colleague and friend in this honorary piece has proven to be very enjoyable for me. Here I am adding new stories to show how important collaboration can be to one's professional and private life.

While I was a graduate student of Bryce DeWitt's at the University of Texas, he invited Chris Isham (from King's College London, UK) and Karl Kuchař (from the University of Utah) to Austin, TX, during the summer of 1974. They were in Austin to discuss which problems in quantum field theory and gravitation needed addressing. I had the luck to sit in on some of those meetings, learning from those great minds. Furthermore, I chose a topic for my PhD dissertation from one of the problems they discussed. Some of the issues were extremely deep, but one, the extension of the Schwinger–DeWitt proper-time regularization method via so-called point-splitting, was something I wanted to do and felt that I could. Mike's name and publications on dimensional regularization techniques also came up at those meetings. This was my first exposure to his research.

In the spring of 1975, I worked continuously on the long and detailed calculations required to express the stress-energy tensor in curved space–time via point-splitting. At times it was clear that hand calculations were potentially too difficult to do accurately. Using computer programs such as FORMAC and Schoonship to do symbolic manipulation of equations via computers was tried, but they were not designed to do or to have exactly what I needed—which was more sophisticated pattern-matching routines. After months of work on the calculations, I noted that the trace of the non-divergent part of the stress tensor expectation value was not zero in those cases where it was classically expected to be zero. DeWitt was disturbed by this, thinking I had made an error somewhere in more than a hundred pages of equations. He told me not to worry about that error until I wrote a later paper (based on my dissertation [10]) for publication and would be able to redo and recheck everything. What I did not know at the time was that Mike and Derek Capper had already found something similar via dimensional regularization [8,11].

After my PhD was done, I left for my first postdoctoral position at King's College London, having been invited there for a year by Isham. It is an understatement to say that that was a wonderful opportunity. The work of Parker, Bekenstein, Hawking and many others had revitalized the excitement in studying black holes and quantum gravity in 1974 and earlier. In 1975–76, the UK became the very centre of work in these areas. At King's, there was Mike, Chris Isham, Paul Davies, David Robinson, Steve Fulling, me and visitors like Stan Deser and Bill Unruh (figure 1). At Oxford, UK, DeWitt was visiting Dennis Sciama, Philip Candelas, David Deutsch and Roger Penrose. At Cambridge, Stephen Hawking, Gary Gibbons, Don Page, Malcolm Perry and others were active. Weekly visits to Oxford and monthly ones to Cambridge allowed discussions unlikely to be found anywhere else at that time.

**Figure 1.**
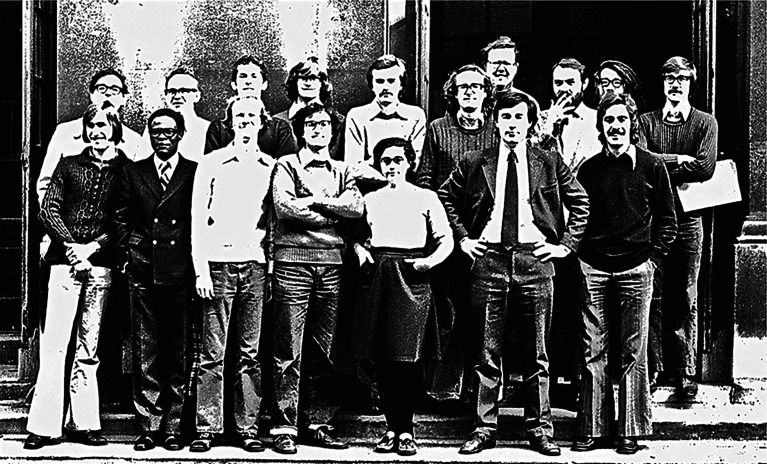
The group at King's College London, 1975–76. Mike is to the right in the front row next to Isham. Steve C is third from the left in the front row with Steve F behind on his right. Deser is behind Isham and Mike; Paul Davies is in the back on Mike's left.

Mike, Steve Fulling and I shared an office at King's. For the first few months, I worked on a paper [12], expanding and checking my dissertation on point-splitting, while trying to work out why the stress tensor had an anomaly. I accepted that DeWitt was right about it having to be zero. Mike and I have told the story [8,9] about how one day we first seriously interacted. He was looking for an explanation of the form of the anomaly he and Capper had found and asked me if I had anything like that in my point-splitting work. I showed him in a paper I was holding that I had, indeed, encountered the same thing and that it was related to the a2-coefficient in the Schwinger–DeWitt method.

We became excited, and Mike immediately walked down the hall with the papers to share the news eagerly with the rest of the group. I smiled at his enthusiasm in seeing that both point-splitting and dimensional regularization gave the same results. I was finally convinced that looking for a way to get rid of the anomaly was the wrong approach and a waste of my time. In fact, it even opened new directions of research.

This all quickly came to a head in two ways: first, by coming to a deeper understanding of the subject through detailed discussions with Mike and Steve Fulling, I was even more certain that the anomaly was there. When Fulling and I went to a talk at Oxford given by DeWitt on quantum field theory in curved space–times, DeWitt eventually reached the point of talking about the trace anomaly and showed how it was zero. I stared in shocked disbelief at his equations on the chalkboard, first thinking we had all made an error, but then I realized that Bryce alone had made an error (a rare event). I looked over at Fulling, who seemed equally puzzled, and I said, ‘That's wrong!’ We gently pointed this out to DeWitt, who did not believe us. But after looking at the board longer he raised a quizzical eyebrow, needing more time to think about it. Eventually, he saw the truth and acquiesced that the anomaly was real.

Second, what further convinced us of the validity, value and structure of the anomaly was the time in 1976 when Fulling and I showed how the trace anomaly in two-dimensional Schwarzschild space–time is equivalent to the existence of Hawking radiation. We also showed that it contributes to the four-dimensional case as well [13].

Mike left for Queen Mary University of London, where he worked on instantons and supergravity. I took up a postdoctoral position at the University of Utah in Salt Lake City, UT, where I spent time extending my scalar field stress tensor calculations to higher spin fields.

After a year, I went to Harvard, Cambridge, MA, at the invitation of Bill Press and Larry Smarr in the physics department and at the Center for Astrophysics. Coincidentally, Mike was invited to Brandeis, Waltham, MA, to collaborate with Stan Deser and his group a few months later. I spent the autumn of 1977 writing up my point-splitting extensions [14]. When Mike arrived, we got together and realized that the anomalies I had been computing for higher spin fields could be used in his work.

We had an unusual working situation. I had randomly chosen to rent an apartment in Waltham but worked in Cambridge, whereas Mike had a place in Cambridge and worked in Waltham. Being a night owl (some called this ‘keeping vampire hours'), I worked until 4 a.m. and did not start working again until noon. As Mike did not have a car, he took the train from Cambridge to Waltham. We soon realized that it was best for him and me to meet around lunchtime at his Brandeis office in Waltham (figures 2 and 3). In the evenings I drove him to Cambridge and did what I needed to do at Harvard—except on Thursdays, when we met there to go to the renowned theory seminars at Harvard or MIT. (I think that Mike might have been a little envious of my sporty new Toyota Celica GT.)

**Figure 2.**
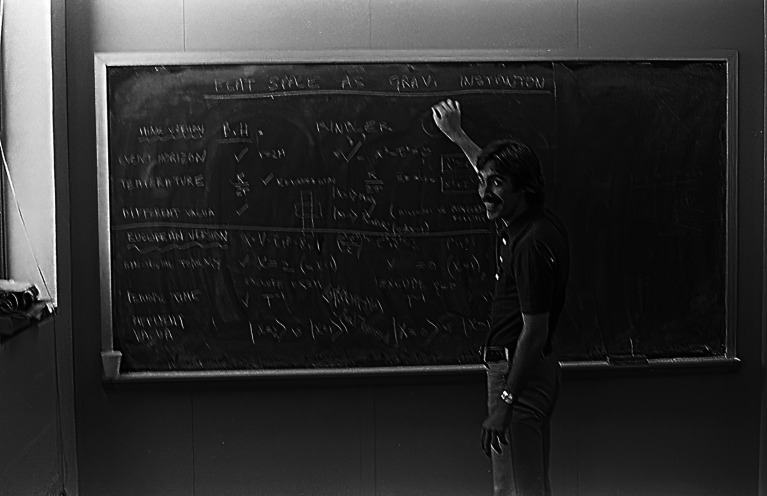
Mike at the blackboard in his Brandeis office, happily talking about our gravitational instanton work.

**Figure 3.**
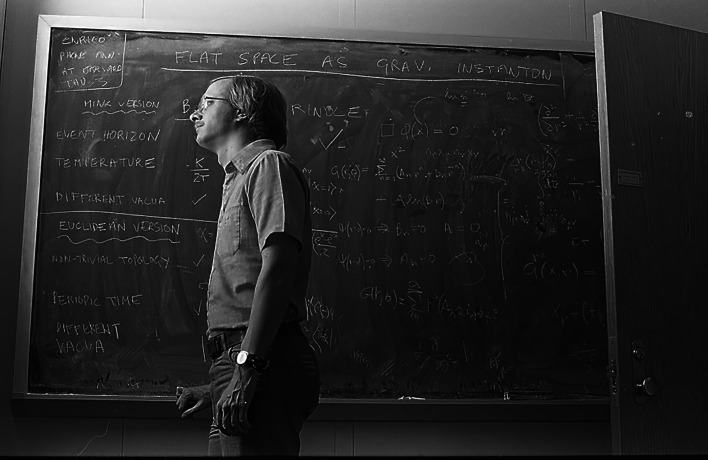
Steve gazing out of the window in Mike's office, probably wondering whether he can get his car started!

Working with Mike was enjoyable because he was so knowledgeable in many areas of physics and had a bit more research experience than I did. At that time he was the ‘idea person’ and I liked being the ‘calculator’, relishing detailed calculating and finding ways to make the calculations perfectly accurate and much faster. There were a lot of people around the world doing quantum field theory in curved space–times and things ‘super’. Some thought that work would surely lead to a ‘theory of everything’ and eventually to a Nobel Prize. Until recently the notions of supersymmetry and string theory (started in those years) dominated theoretical research.

Mike and I started working day and night on ‘super’ things. We began to see relationships between the Schwinger–DeWitt coefficients and topological aspects of quantum field theory in the work of Atiyah, Singer and others. We also looked at the axial anomaly in addition to trace or conformal anomalies. We wondered about cancellations or combinations of anomalies in supergravity theories. Might we be able to remove the divergences that plagued all such theories?

Having colleagues like Deser, Grisaru and Ovrut at Brandeis as well as Weinberg and Bott at Harvard gave us more ideas and support. Mike has written about our meeting at Harvard with Weinberg [8], who was interested in our 2 + ε quantum gravity work. Being invited to chat in the office of such a luminary was both scary and encouraging. Weinberg was about a year away from his Nobel Prize then. (Years later in 2018 I was pleased when Weinberg told me in Austin that he remembered the subject of our talk that afternoon.) Similarly, when we asked to see Bott to find out more about index theorems and related subjects, we were happy to be taken seriously by such a great mathematician.

An incident with Mike at Harvard that I remember vividly had to do with a seminar given by a graduate student presenting his research topic. During his talk, the student mentioned work he had done that overlapped very closely with Mike's and my work. However, when questioned he refused to admit having knowledge of our papers. I didn't believe him and started to become annoyed, gripping the seat with my right hand until my knuckles turned white. This was not my normal reaction to such an affront, but this fellow seemed insufferable. I must have looked like I was going to get angry when Mike reached over and tapped my arm to calm me down. He knew how to handle such things diplomatically, and I learned something new that day.

As we continued to write papers, we found that producing them was getting tedious. We would work very late, often talking on the phone until after midnight, seven days a week. While discovering ‘super’ things, we found that we were in quite fierce competition with others in Europe. We knew that we might ‘get scooped’, so we had to quickly get the work done, written up and sent off. Typewriting long papers with lots of equations at that time was not easy even with help from our departments, so I mentioned this to Bill Press. His polymath mind had an instant solution: he had a minicomputer (a Data General Nova 3) with a typesetting system that could help to do and format equations. We were free to use it at night in his office if we wanted. My past programming experience and ability to type were put to work producing our papers. We would routinely work at Brandeis, drive to Harvard and write the papers at the terminal until late at night. I have always been grateful to Bill for his support with this project.

All of this was more exciting and exhilarating than I could have ever imagined despite the stress, pressure and worry. However, since I was learning so much with Mike and feeling so pleased about doing good work, nothing else mattered. Even the February blizzard in 1978 did not dampen our excitement. The winter weather did present some issues, though. As I did not have a parking permit for Brandeis, I had to use a parking lot far away. The weather was extremely cold, and the lock on my car door was often frozen. So, Mike and I had to carry hot water all the way from the building to the car to defrost the doors day after day. I suspect an experimental physicist would have seen the folly of our attempts and solved the problem far more efficiently.

Eventually, Mike headed back to Imperial College London, UK, and I went to the new Institute for Theoretical Physics (ITP) in Santa Barbara, CA, for a year. It was not long before there was a conference at SUNY-Stony Brook that Mike and I attended and simultaneously finished some new work. Our penultimate paper [6] was written during the two-week conference. We had read two new papers from a group in the UK, checking their work against ours and finding significant differences. So, we wrote up a new paper challenging their work and presenting our (correct) results in what might be called an irreverent, mischievous way. Some of our colleagues questioned our wording, but we insisted on keeping it. Down the road, this paper apparently offended certain people and got me in some ‘hot water’ (which I will not elaborate on here).

During my second semester at ITP (now Kavli ITP), Mike, Bryce DeWitt, Gary Gibbons and Martin Roček were visitors in Santa Barbara. In my seventh collaboration with Mike, Gary and Martin joined us [7].

Mike left the Institute to go back to Imperial, and I went to the University of North Carolina (UNC) at Chapel Hill, learned about teaching in the classroom and started to move in a new direction away from ‘super’ things. Mike continued to contribute groundbreaking work in supergravity, superstrings and M-theory for years to come.

It became clear that any further work I would do in point-splitting would need to be computer-aided. The work on the computer at Harvard had made me appreciate programming again. I started off into the world of scientific software development, which led to a lot of consulting and ultimately to three software companies over the following 40 years. These companies, stimulated in part by past work with Mike, still exist and are doing well.

In the early 1980s, Mike and I saw each other again in the UK and in Austin. By that time, he was deeply involved in super theories and in strong competition with people trying to initiate the next great advance. It was clear to me that this world of competition was not where I wanted to be. Mike's stories about the ruthlessness in his work world made me want to avoid such experiences. He, however, was capable of being a leader in this competitively tough world of physics. Over the years, I watched him make major progress in many areas and saw him on and off in London or Chapel Hill.

After 30 years of physics-related computing work, including the development of the MathTensor software with Leonard Parker, I returned to physics at UNC. In August 2015 I reunited with many of the 1970s group at the Hawking Radiation Conference in Stockholm, Sweden. For me, getting back together with Mike, Steve Fulling, Paul Davies and others was fantastic (figures 4 and 5). It felt as though no time had passed.

**Figure 4.**
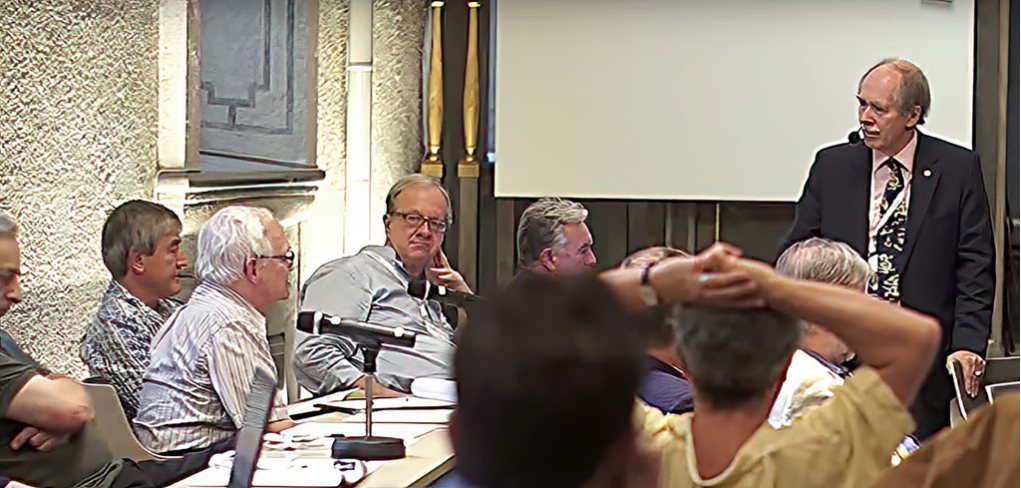
The King's College group reunion at the Hawking Radiation Conference in Stockholm in August 2015 listening to ‘t Hooft: Davies, Fulling, Christensen and Mike. All of us greyer, but still enthusiastic. (Online version in colour.)

**Figure 5.**
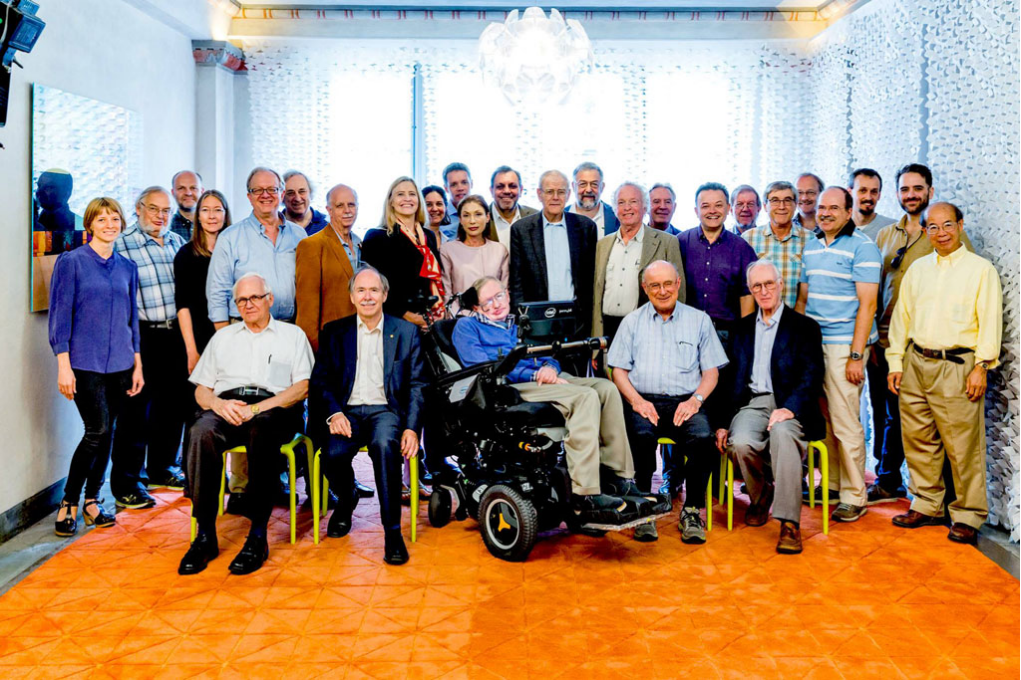
The conference photo in Stockholm with the old guard of quantum field theory in curved space–times as well as the new generation. (Online version in colour.)

During my active life in physics, many valuable experiences came directly or indirectly from working with Mike. I have much to thank him for.

## Data Availability

This article does not contain any additional data.
